# Complex auditory regularity processing across levels of consciousness in coma: Stage 1 Registered Report

**DOI:** 10.1093/braincomms/fcae466

**Published:** 2024-12-21

**Authors:** Andria Pelentritou, Jacinthe Cataldi, Frederic Zubler, Manuela Iten, Matthias Haenggi, Nawfel Ben-Hamouda, Andrea O Rossetti, Athina Tzovara, Marzia De Lucia

**Affiliations:** Department of Clinical Neurosciences, Lausanne University Hospital (CHUV), University of Lausanne, 1011 Lausanne, Switzerland; Department of Clinical Neurosciences, Lausanne University Hospital (CHUV), University of Lausanne, 1011 Lausanne, Switzerland; Department of Neurology, Spitalzentrum Biel, University of Bern, 2502 Biel, Switzerland; Department of Intensive Care Medicine, Inselspital, Bern University Hospital, University of Bern, 3010 Bern, Switzerland; Institute of Intensive Care Medicine, University Hospital Zurich, 8091 Zurich, Switzerland; Department of Adult Intensive Care Medicine, Lausanne University Hospital (CHUV), University of Lausanne, 1011 Lausanne, Switzerland; Department of Neurology, Lausanne University Hospital (CHUV), University of Lausanne, 1011 Lausanne, Switzerland; Institute of Computer Science, University of Bern, 3012 Bern, Switzerland; Department of Neurology, Center for Experimental Neurology, Bern University Hospital (Inselspital), 3010 Bern, Switzerland; Department of Clinical Neurosciences, Lausanne University Hospital (CHUV), University of Lausanne, 1011 Lausanne, Switzerland; Centre for Biomedical Imaging (CIBM), 1011 Lausanne, Switzerland

**Keywords:** EEG, consciousness, coma, local global paradigm, multivariate decoding

## Abstract

A key question for the scientific study of consciousness is whether it is possible to identify specific features in brain activity that are uniquely linked to conscious experience. This question has important implications for the development of markers to detect covert consciousness in unresponsive patients. In this regard, many studies have focused on investigating the neural response to complex auditory regularities. One noteworthy example is the local global paradigm, which allows for the investigation of auditory regularity encoding at the ‘global’ level, based on the repetition of groups of sounds. The inference of global regularities is thought to depend on conscious access to such complex auditory stimuli as mostly shown in chronic stages of disorders of consciousness patients. However, whether global regularity encoding can identify covert consciousness along the consciousness spectrum including earlier stages of these disorders remains controversial. Here, we aim to fill this gap by investigating whether the inference of global auditory regularities can occur in acute coma, in the absence of consciousness, and how this may be modulated by the severity of the patients’ clinical condition and consciousness level measured using the Full Outline of UnResponsiveness (FOUR) score. We will acquire 63-channel continuous electroencephalography to measure the neural response to global auditory regularity in comatose patients (*N* = 30) during the first day after cardiac arrest, when patients are unconscious, sedated and under normothermia, and during the second day (with reduced or absent sedation and body temperature control). We hypothesize that global regularity encoding will persist in the absence of consciousness independent of patient outcome, observed as above chance decoding of the neural response to global regularities using multivariate decoding analyses. We further hypothesize that decoding performance will positively correlate with the FOUR score, which indexes consciousness level, and typically improves between the first and second day after coma onset following cardiac arrest in patients with favourable outcome. In an exploratory analysis, we will also evaluate whether global regularity encoding may be influenced by the patients’ clinical management, specifically sedation, also shown to affect global deviance detection. Our results will shed light on the neurophysiological correlates of complex auditory regularity processing in unconscious patients and on the link to residual levels of consciousness during the underexplored state of coma upon the first days after cardiac arrest.

## Teaser

Pelentritou *et al*. plan to investigate whether global auditory regularity encoding persists in acute coma after cardiac arrest. Using EEG and multivariate decoding analyses, they will examine correlations with consciousness levels and the impact of sedation, shedding light on auditory processing and residual consciousness in the early stages of coma.

## Graphical Abstract

**Graphical Abstract:**
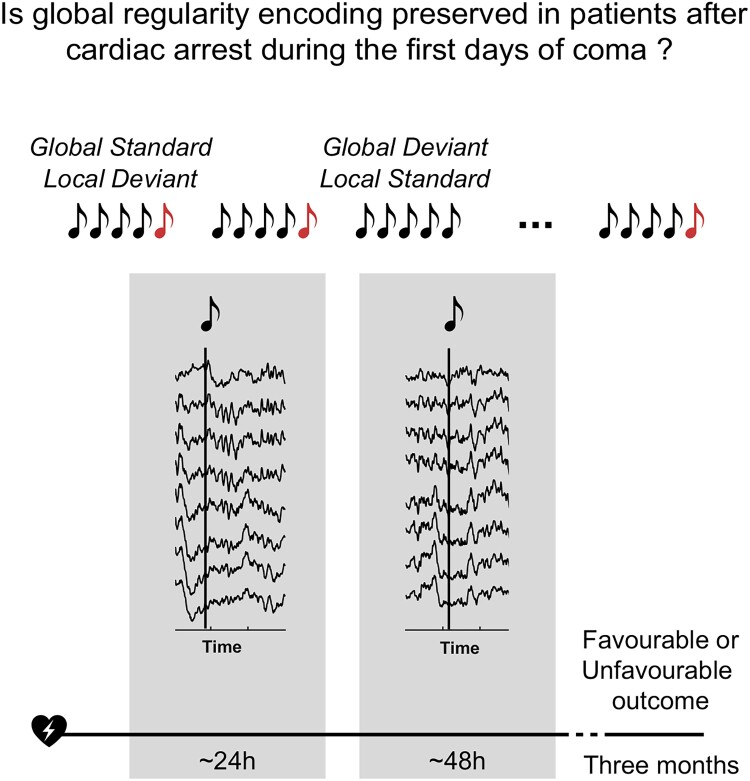

## Introduction

Uncovering the neural underpinnings characterizing conscious and unconscious processing of sensory stimuli lies at the forefront of neuroscientific exploration.^1,2^ This is an essential step for the development of clinical markers that can inform on the level of consciousness in unresponsive patients, based on the identification of the key features of brain activity associated to conscious experience. Currently, consciousness level assessment requires multiple lengthy examination sessions which frequently result in an uncertain diagnosis with an estimated misclassification rate up to 40% in chronic patients.^3^ The development of quantitative markers based on neuroimaging and electrophysiological recordings can provide complementary information to the outcome of standardized clinical examinations and help reduce the uncertainty in consciousness level assessment.^4,5^ The majority of experimental paradigms, whether based on the neural responses to sensory stimuli, resting state activity or perturbational approaches (reviewed in^5,6^), have so far mostly focused on the prognosis and diagnosis of patients with chronic disorders of consciousness (DoC; >28 days).^7-9^ Whether experimental paradigms and resulting biomarkers developed in the context of chronic DoC may also be appropriate for studying preserved neural processes in other states of altered consciousness such as the early stages of coma remains largely unresolved.^5^ Our study aims to fill this gap by investigating the degree of preserved complex auditory regularity encoding in comatose patients during the first days after cardiac arrest, using the well-established local global paradigm,^10^ and to explore the relationship between auditory encoding and the patients’ clinical features and consciousness level.

The local global paradigm is based on the repetition of groups of sounds with two regularity types: first, at the level of the single sound repetition (local regularity) and second, based on repeating a group of five sounds (global regularity).^10^ By measuring the EEG responses to local and global regularities, it has been possible to infer on the degree of auditory regularity encoding across varieties of conscious and unconscious states, in healthy and clinical populations.^10-18^ The encoding of local regularity is typically considered an automatic process that can occur in the absence of consciousness.^19^ On the other hand, global regularity encoding implies the formation of a memory trace over the temporal scale of the group and may require conscious perception of the auditory regularity.^10,11^ Studies investigating global regularity processing reported a late EEG differential response—typically after ∼300 ms post-stimulus onset—when comparing global standard and global deviant stimuli in healthy volunteers who could detect the global auditory deviants as well as DoC patients with—presumably—covert consciousness.^10,14^ This response is often identified as the P3b component,^13,14,20^ an EEG component^21^ that has been proposed as a marker of conscious access to sensory stimuli in the context of the Global Workspace Theory.^22^ However, evidence of preserved P3b responses upon auditory processing in subliminal perception,^23^ altered consciousness states like sleep^24-26^ and DoC,^27-31^ has cast doubt on whether this electrophysiological marker can be uniquely associated with conscious experience.^1^ Indeed, these studies suggest that experimental paradigms testing brain responses to sensory stimuli, traditionally associated with the P3b component, can be elicited even outside of conscious experience.^1^ Similarly, and specific to the local global paradigm, an account of global regularity encoding in comatose patients after cardiac arrest during the first day after coma onset,^15^ suggests that such complex auditory processing may occur in deep unconscious states. This finding sparked a fruitful yet unresolved debate on the mechanisms of complex auditory stimulus processing.^32-36^ It also highlights the importance of understanding how processing global regularities may depend on the severity of the patient’s clinical condition and on how their clinical condition evolves over time. Such an exploration will address whether global regularity encoding can serve as an experimental marker for covert consciousness detection across a variety of clinical conditions.

Here, we aim at investigating and validating the conditions under which the neural processing of global regularities can occur in the absence of consciousness^15^ and at assessing whether the degree of preservation of global regularity encoding depends on the severity of the DoC patients’ clinical condition and consciousness level. To this aim, we will investigate global regularity encoding in patients during the early hours after coma onset, i.e. during the first and second day following cardiac arrest when patients are typically in a deep unconscious state. In more detail, in this cohort of unconscious patients, we hypothesize the following:

1. Global regularity encoding can be detected during the first day of coma after cardiac arrest when patients are sedated and under body temperature control (normothermia, < 37°C). During the first day of coma after cardiac arrest, conscious access to external stimuli is considered extremely unlikely, as standardized clinical examination typically provides scores indicative of a deep unconscious state.^5^ This hypothesis is based on our seminal work, which revealed a significant decoding of the EEG responses to global standards versus global deviants in five comatose patients on the first day after cardiac arrest.^15^ As a sanity check, the analysis of global regularity encoding will be complemented with that of local regularity encoding in the same cohort.
2. The degree of preservation of global regularity encoding will be dependent on the severity of the patient’s clinical condition and consciousness level. We hypothesize a higher preservation of global regularity encoding (observed as higher multivariate decoding performance when contrasting global standard and global deviant stimuli) in patients with less severe clinical condition. In order to evaluate this hypothesis, we will investigate the correlation between the EEG-based global decoding results and the Full Outline of UnResponsiveness score (FOUR score^37^), separately for the first and second day after coma onset. While the FOUR score represents an indirect measure of the level of consciousness as it is based on a behavioural assessment, it represents the gold standard for the clinical evaluation of acute and intubated coma patients.^4,37^ Herein, it will therefore be used as a proxy for the degree of clinical severity and level of consciousness.^38-40^ We will perform this correlation analysis separately for the first and second day in order to include a homogeneous cohort of patients under similar clinical management conditions. In addition, the second day after coma onset is characterized by FOUR scores typically more variable compared with the first day and with higher values in patients who will eventually recover (see for example Supplementary Table 2 in Pelentritou *et al*.^41^). Having a range of FOUR score values will maximize the possibility of uncovering potential correlations between the strength of the global decoding and the FOUR score results.
3. Global regularity encoding will be more prevalent in patients with favourable than in patients with unfavourable outcome across the two recording days. This hypothesis is based on previous studies in patients with DoC evaluated after days or weeks after coma onset, which demonstrated a higher prevalence of global regularity encoding in patients with favourable outcome.^13,14,20^ Patient outcome will be dichotomized to favourable or unfavourable [based on the best cerebral performance categories (CPCs^42^) score; see the ‘Patient recruitment and description’ section in Materials and methods], and global regularity decoding performance will be compared across the two populations. We will additionally explore whether the encoding of global regularities correlates with the CPC scores (ranging from 1 to 5), in order to identify whether the gradient of auditory processing can relate to coma evolution beyond a binary outcome definition as favourable or unfavourable.
4. In an exploratory analysis, we will investigate whether global regularity encoding will be influenced by quantifiable clinical factors, namely administered sedative agents, since anaesthetic administration has also been shown to modulate global deviance detection.^12,18^ This will in turn identify additional factors at play upon assessing the relationship between global regularity encoding and patients’ clinical characteristics and will aid in clarifying the heterogeneity of the EEG results across patients, as observed in our previous study where only five out of 24 patients exhibited accurate global regularity encoding.^15^

## Materials and methods

### Ethical approval

Approval for the study (Project-ID: 2023-00271) was obtained by the local ethics committee (La Commission Cantonale d’Ethique de la Recherche sur l’Etre Humain) in accordance with the Helsinki Declaration.

### Sampling plan

This analysis was based on code and recommendations on sample size estimation for multivariate decoding derived from the Fieldtrip toolbox.^43^

#### Comatose patients

We assumed a binomial distribution for computing the probability of obtaining K significant decoding results (i.e. *success*) in *N* recordings. The probability of observing *a priori* significant decoding when classifying single trials in response to global standards and global deviants was estimated based on our previous study where we obtained significant decoding results in five out of 24 recordings during the first day of coma, i.e. *P* = 0.21.^15^ We then simulated the same experiment *n* = 5000 times using a binomial distribution where *a priori* we defined the total number of tests *N* (or recordings in our application) and *P* the *a priori* probability of success. The *n* simulations were repeated for each value of *N*, a total number of tests, with *N* ranging from 4 to 30. In each simulation, we computed the number of success *K*′, resulting in an array of *n* values for each value of *N*. Next, we calculated the probability P_H0 of obtaining up to *K*′ success for each experiment realization under the alternative hypothesis H_0_ of a zero *a priori* probability of obtaining a success. The power was computed based on the number of times the probability under the alternative hypothesis was less than a predefined threshold of α=0.05: Power $(N)=\sum_{i=1}^{n}\;(P\_H0_{i}(N)\;<\;\alpha )/n$. The simulation showed that a power of 0.95 can be obtained with a sample size of 13 patients, corresponding to an average value of success <K’> = 2.7 across the 5000 realizations. In this study, we adopted a more conservative approach by planning 30 comatose patient recordings in order to maximize the number of patients available for the additional acquisition of a second day recording and to account for possible technical issues resulting in recording exclusion.

#### Healthy controls

In our previous study, a cohort of healthy controls was also included and significant decoding when classifying the neural responses to global standards and global deviants at the single-trial level was obtained in two out of 10 passive healthy control recordings, i.e. *P* = 0.20 (and in four out of 11 healthy participants during an active task).^15^ Here, we performed the same analysis as described above for comatose patients, which showed that a power of 0.95 can be obtained with a sample size of 13 passive healthy controls. Therefore, we will recruit 15 healthy controls, to account for potential technical issues or excessive artefacts that may result in participant exclusion.

### Patient recruitment and description

We will recruit patients during the first day (up to 28 h), and when possible, during the second day (up to 56 h) after cardiac arrest at the intensive care units of the University Hospitals of Lausanne and Bern in Switzerland. Patients will be in a deep unconscious state (using an operative definition based on the Glasgow coma scale^44^ and FOUR score,^37^ detailed below in the inclusion criteria) after exclusion of patients with the so-called coma mimic (paralysis, psychogenic disorder). Patients will be treated according to local standard of care, including analgo-sedation with midazolam, propofol and/or fentanyl (as well as neuromuscular blockade if needed^45,46^) and control of body temperature with normothermia (<37°C). Patients’ clinical management will be decided solely based on clinical assessment and will not be affected by the present study.

Clinical and demographic information, including age, sex, time to return of spontaneous circulation , first recorded cardiac rhythm, as well as body temperature and sedative agents and dosage during the EEG acquisition will be additionally collected. In addition, we will collect the results of the neurological examinations such as brainstem reflexes, pupillary reflexes, corneal reflexes and motor reactivity and visually characterize the EEG (background reactivity, discontinuity and presence of an epileptiform activity, in accordance with the ACNS recommendations^47-49^), when available. Of note, patients will not be excluded from the study if some of this information, clinical, demographic or as a result of the neurological assessment, will not be available. Individuals who remain unconscious will be assessed according to a protocol based on the European Resuscitation Council and European Society of Intensive Care Medicine.^50^

A follow-up of the patients will be performed at 3 months after cardiac arrest (routine phone call with a semi-structured interview) as assessed by the CPC^42^ and modified Rankin scale (mRS,^51^) when available. Patient outcome will be defined based on the best score within 3 months after cardiac arrest considering the CPC values, evaluated at 3 months, the outcome at the hospital discharge and neurological examination during hospitalization.^41,52^ Patient outcome will in turn be dichotomized to favourable (CPC of 1–2) or unfavourable (CPC of 3–5).

For recordings to be performed within the first day of coma onset, the inclusion criteria will be:

- Age ≥ 18 years
- Admission and treatment in an intensive care unit after cardiac arrest
- Glasgow Coma Scale of a total less or equal to 7 out of 15 with eye response of 1 out of 4, verbal response of 1 out of 5 and motor response inferior or equal to 5 out of 6
- FOUR score of a total less or equal to 8 out of 16 with eye response of 0 out of 4, motor response between 0 and 3 out of 4, brainstem reflexes between 0 and 4 out of 4 and respiration between 0 and 1 out of 4
- Informed consent obtained by a proxy (family member, legal representative), completed subsequently by the patient consent if capable of discernment within 3 months.

The exclusion criteria will be:

- High likelihood of needing a surgical intervention or an invasive diagnostic procedure within the following 48 h after cardiac arrest according to the treating physician (as this would prevent access to the patient).
- High likelihood of withdrawal of life-sustained therapy within the first 48 h after admission.
- Deafness before the acute brain injury.
- Logistic reasons (e.g. unavailability of research team to perform the recordings or resources allocated to another patient).
- Presence of a large cerebral lesion other than post-hypoxic encephalopathy (e.g. subarachnoid haemorrhage, intracerebral haemorrhage and large ischaemic stroke).

Acquisition of a second day recording will not be performed in cases where patients recover or pass away rapidly after the first day recording or when access to the patient is prevented by urgent medical intervention or for logistic reasons, such as unavailability of the experimenter and/or of the necessary equipment. Patients for which a second day recording will not be available will be included only in the first day analysis.

### Healthy participant recruitment and description

Control participants will be recorded in order to estimate the decoding performance for global regularities in a healthy cohort. We will recruit 15 healthy control participants between 50 and 62 years old. The age range was selected to match the age range of comatose patients after cardiac arrest, which showed significant global regularity encoding in our previous study.^15^ Healthy controls will be recruited using flyers and participant recruitment databases. Control participants will be asked to lay comfortably in an inclined chair and keep their eyes closed while the passively listen to the sounds. The EEG signals will be monitored online by experienced sleep scoring researchers (J.C. and A.P.) to ensure control participants are awake during the recordings.

### Experimental design and data acquisition

The same experimental paradigm will be implemented in patients on the first and second day after coma onset as well as in healthy control participants.

**Figure 1:**
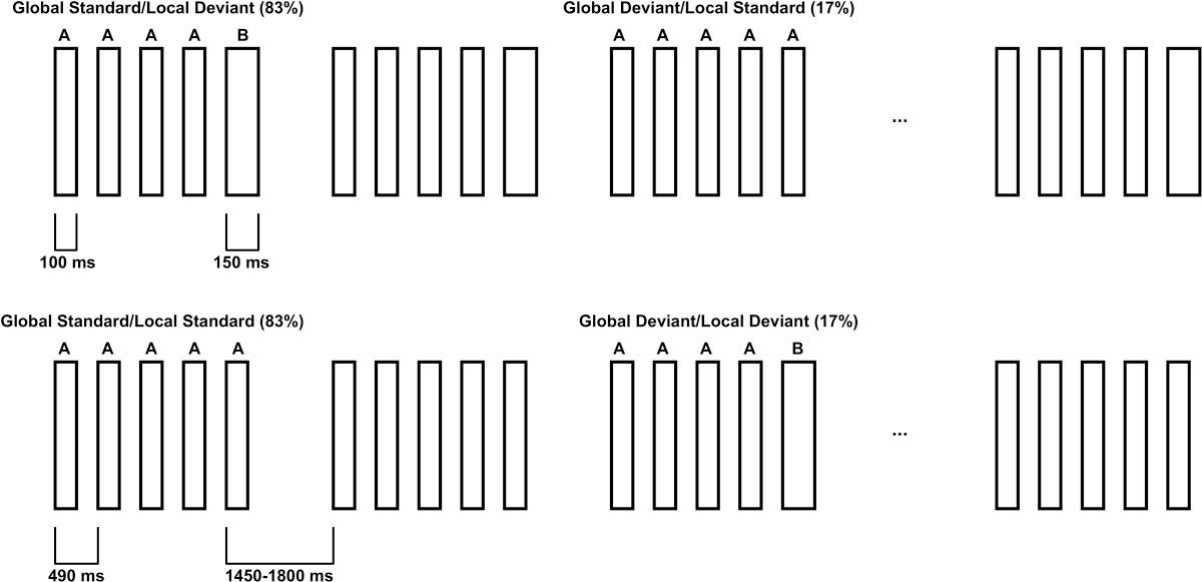
**Experimental protocol.** The proposed experimental paradigm will include four block types, two of which are shown here. Individual sounds will be of 100- or 150-ms duration (narrow and wide rectangles, respectively) and will be administered in series of five sounds. The five stimuli can have either identical or different duration and play the role of standard or deviant stimuli at the global or the local level. In the other two blocks of the experiment, the 100- and 150-ms duration individual sounds will be swapped with respect to what is shown here. Each block type will be repeated twice resulting in a total of eight administered blocks for each patient. The experimental design will be similar to the one adopted in our seminal study^15^ and is based on^10^.

Continuous 63-channel EEG (g.HIamp, g.tec medical engineering, Graz, Austria) will be acquired at 1200 Hz while administering auditory sequences via in ear headphones (Fig. 1) in patients after cardiac arrest (*N* = 30) and in healthy controls (*N* = 15). Additional ECG electrodes will be attached to the patients’ chest, a vertical EOG electrode will be attached below the right eye, and a horizontal EOG electrode will be attached to the left outer canthus. Impedances of all active electrodes will be kept below 50 kΩ. All data will be recorded with online 0.1–100 Hz band-pass filters.

Auditory sequences will follow the local global paradigm.^10,15^ This paradigm will be based on the repetition of groups of sounds with short and long duration.^15^ Individual sounds will be binaural synchronous pure tones of 100 ms (A) or 150 ms (B) duration (Fig. 1). Four different series of sounds will be administered, either consisting of five identical sounds (AAAAA or BBBBB) or of four identical sounds and one last different sound (AAAAB or BBBBA). By repeating the series of five sounds, we will induce a global regularity (in Fig. 1 this corresponds to repeating the series AAAAB in the first line and AAAAA in the second line). Local regularity will instead be investigated within the series of sounds where the last sound differs from the first four (in Fig. 1 this corresponds to A versus B in the first series of the first line and in the third series of the second line). In the first block type (Fig. 1, first line), the AAAAB series will be the global standard and the AAAAA series will be the global deviant, while in the second block type (Fig. 1, second line), the global standard and deviant series will be reversed (i.e. AAAAB and AAAAA, respectively). The third and fourth blocks will follow the same structure; however, A and B individual sounds will be reversed (i.e. block 3: global standard: BBBBB, global deviant: BBBBA; block 4: global standard: BBBBA, global deviant: BBBBB). The fixed time interval between the onset of individual sounds will be 490 ms and the variable inter-trial interval across series of sounds will range between 1450 and 1800 ms. We will present 120 series of sounds in each block, specifically 100 global standard series and 20 global deviant series. Finally, the entire experimental paradigm will consist of two repetitions of each block type in a semi-randomized order.

### Proposed analysis pipeline

**Figure 2:**
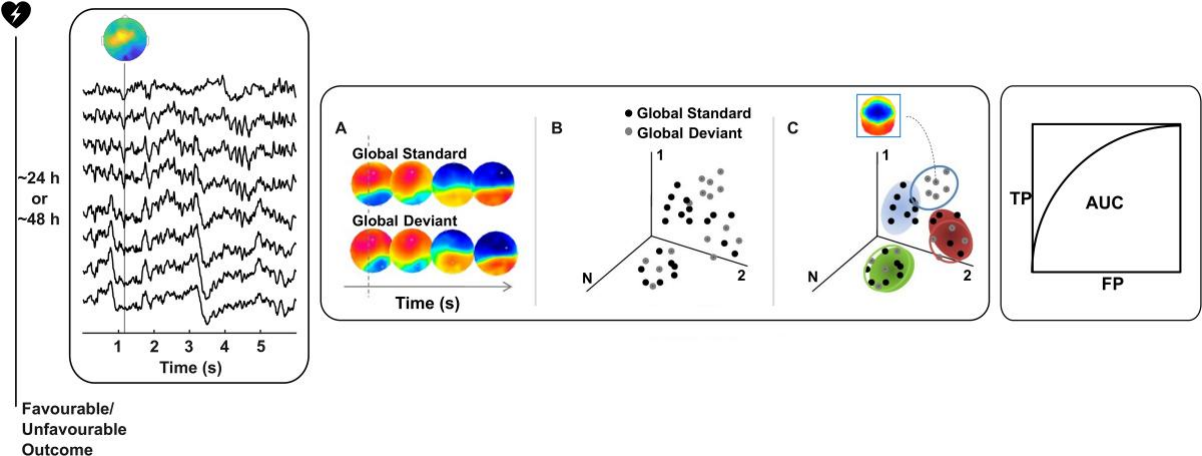
**Main steps of data collection and data analysis pipeline.** Continuous electroencephalography recordings will be acquired, while the local global paradigm (see Fig. 1) is administered in comatose patients during the first (∼24 h) and second (∼48 h) day after coma onset following cardiac arrest (*left* panel). The voltage–topographic response to global and local standard and deviant sounds will be used as input in a multivariate decoding analysis using a single-trial topographical analysis (*middle* panel: A, B, C) or using support vector machines with the goal of classifying standard and deviant sounds. The performance of the multivariate decoding algorithms will be measured based on the AUC (*right* panel). The outcome of the comatose patients will be assessed at 3 months after coma onset using the best CPC scores and classified as favourable (CPC of 1–2) or unfavourable (CPC of 3–5). The relationship between decoding performance and patient outcome as well as additional clinical features will be assessed. TP, true positive; FP, false positive.

The same analysis will be performed for the first day and second day comatose patient recordings as well as the healthy control recordings. We will assess global regularity encoding at the single recording level using multivariate decoding (Fig. 2). We expect significant decoding performance when classifying single-trial EEG responses to global standard and global deviant sounds in at least one-fifth of coma patients irrespective of their outcome and in at least one-fifth or more healthy controls.^15^

#### Preprocessing

Continuous raw EEG data will be band-pass filtered (0.1–40 Hz). Artefact rejection in the continuous EEG will be performed by applying independent component analysis as implemented in EEGLAB^53^ and Fieldtrip^43^ and removing any components resulting from artefact activity. EEG analyses will be based on the data extracted from −100 to 500 ms relative to the onset of the last sound in the group of five sounds. A semi-automated approach for artefact rejection and channel interpolation will be implemented in Fieldtrip.^43^ Next, we will perform two main analyses for quantifying the global and local effect at the single recording level. We will assess the decoding performance in classifying EEG responses to global standard and global deviant sounds and EEG responses to local standard and local deviant sounds.

#### Multivariate decoding analysis

Separately for each recording, we will apply multivariate decoding to the global standard versus the global deviant (for the global effect) and local standard versus local deviant (for the local effect) using the single-trial EEG responses (Fig. 2, left panel). The procedure will be divided into a cross-validation (CV) and test phase, based on 80 and 20% of the available trials, respectively, per EEG recording. The CV phase aims at model training and parameter optimization using ∼80% of the available trials. In the test phase, the algorithm’s performance will be assessed on a different set of trials to those used in CV. The decoding performance will be measured based on the Area Under the receiver operating Characteristics (AUC) and compared to the distribution of decoding results obtained from training the model on the datasets with permuted labels, with a large number of permutations (*N* ≥ 100) (Fig. 2, right panel) (see ‘Statistical analysis plan’).

The first multivariate decoding analysis to be performed will be the single-trial topographical analysis (STTA^54^), based on previous work identifying deviance detection in coma,^15,55,56^ in which the voltage topographies at the single-trial level are modelled using a mixture of Gaussians (GMM^57^). The analysis of the single-trial EEG responses using the STTA algorithm allows for uncovering the time interval or set of intervals and topographies that carry the most discriminative information in response to the different experimental conditions of interest.^58-60^ The GMM parameters’ estimation will be based on the ensemble of instantaneous voltage topographies, from all latencies and trials, each of them normalized by the instantaneous global field power (Fig. 2, middle panel, A). The decoding algorithm takes into account the mean voltage topographies (template maps) for each Gaussian in the GMM and the period of time at which these template maps mostly differ between the two conditions of interest (Fig. 2, middle panel, B, C).^54^ The optimal number of Gaussians will be estimated in the CV dataset in order to maximize the AUC. Following this parameter selection, the test phase will consist of evaluating the corresponding multivariate decoding algorithm in an independent set of data.

In addition to this multivariate decoding based on STTA, we will investigate the local and global effect by classifying the EEG single-trial responses using a support vector machine (SVM) algorithm.^61^ The aim is to evaluate the consistency of the decoding results across different decoding strategies and importantly, to explore the different strengths of the STTA-based and the SVM-based algorithms in decoding EEG responses to stimulus classes. SVM classification is a commonly employed supervised learning technique allowing for separating classes based on a hyperplane with optimized margins in a high-dimensional space (see^62-64^ for similar applications). The advantages of this approach are, amongst others, the capacity to withstand high-dimensional data and a good performance when both linear and non-linear relationships exist between predictive variables and target labels.

The inputs to the SVM classifier within each trial will consist of the concatenated EEG vector with voltage values at each channel and time-point resulting in a vector of dimensions #channels x #data points. The classifier will provide a decoding value for each trial with the advantage of exploiting all available data. Hence, accurate classification will be achieved when the differential EEG responses to standard and deviant sounds will be both long-lasting over time and spatially distributed across electrodes. In addition to this analysis, we will also consider a time-point by time-point decoding by repeating the same steps as above, now applied to the vector of voltage measurements across channels at each time-point. This second strategy has the advantage of providing a time-resolved representation of the differential response to standard and deviant sounds.

In the CV dataset only and separately for the global and local trial comparisons, we will test linear and non-linear (radial basis function) kernels for achieving optimal decoding performance. The CV phase in SVM classifier training aims at identifying the best possible kernel parameters, regularization parameter *C* and radial width γ, to enable highest decoding performance. Following optimization of the SVM parameters, we will assess the decoding performance and its significance based on the AUC evaluated in the test set using permutation analysis (see ‘Statistical analysis plan’).

To complete the decoding analysis for both algorithms, we will additionally assess the classification performance when the sub-averages of the EEG single trials across 2 and 3 randomly selected trials are considered. This step has the advantage of increasing the signal-to-noise ratio while decreasing the number of available samples and typically provides better results compared with when single trials are used.

### Statistical analysis plan

#### Multivariate decoding analysis

Permutation testing (100 permutations) will be implemented to test the performance of the STTA and SVM analyses, wherein the AUC will be considered significant if it outperforms the ones obtained with permuted labels as assessed by a Wilcoxon signed rank test (*P* < 0.05).

#### Level of consciousness and decoding results

Pearson’s correlations (*P* < 0.05) will be used to measure the relationship between decoding performance for global regularities and the clinically assessed consciousness level, expressed as the AUC and the FOUR score, respectively.

#### Patient outcome and decoding results

Two-sided Fisher’s exact tests (*P* < 0.05) will assess the relationship between decoding performance for global regularities and patient outcome by contrasting the number of patients which exhibited global regularity encoding based on the AUC within the favourable and unfavourable outcome patients. In addition, Pearson’s correlations (*P* < 0.05) will be used to investigate the degree of correlation between the decoding performance for global regularities and the different patient outcome scores (CPC of 1–5).

#### Clinical and demographic features and decoding results

To quantify the potential influence of patient demographic information and clinical management on the decoding performance, we will statistically compare sedative agents, sedation concentrations, body temperature, EEG clinical features and other demographics between patients for which global standard and global deviant classification was possible against patients for which it was not, independent of patient outcome. To do so, we will utilize non-parametric Kruskal–Wallis analyses of variance which allow for unbalanced and not-normally distributed populations to be statistically compared.

In order to further investigate the impact of the patients’ clinical management on global regularity encoding, we will compute logistic regression models to test the probability of observing a high decoding value for global regularity based on the infusion rates of the different sedative agents commonly administered. Specifically, we will use generalized linear regression models and compute separate models with fixed-effects terms for each administered sedative infusion rate (propofol, fentanyl, midazolam). Models testing the interaction between the fixed-effects terms will also be generated. The response term (i.e. dependent variable) will be the decoding performance expressed as the AUC value. The models that best describe the decoding values will be selected using forward selection based on the adjusted R^2^ values^65^ as well as based on the Akaike information criterion^66^.

## Data Availability

Anonymized raw data from comatose patients will be deposited and become publicly available repository upon submission of the Stage-2 pre-registered manuscript. Any custom-made scripts used in the analysis will be deposited on the publicly available repository of our laboratory: https://github.com/DNC-EEG-platform/.
